# Effectiveness of immunological agents in non‐small cell lung cancer

**DOI:** 10.1002/cnr2.1739

**Published:** 2022-10-26

**Authors:** Akhil Rekulapelli, Lucas E. Flausino, Gayatri Iyer, Rajesh Balkrishnan

**Affiliations:** ^1^ Department of Public Health Sciences University of Virginia School of Medicine Charlottesville Virginia USA; ^2^ Faculdade de Medicina Universidade de São Paulo São Paulo Brazil; ^3^ Department of Pharmaceutical Sciences and Technology Institute of Chemical Technology Mumbai India

**Keywords:** adoptive T cell therapy, cancer, cancer vaccines, immunotherapy, monoclonal antibodies, NSCLC, oncolytic viruses

## Abstract

**Background and aim:**

Non‐small cell lung cancer (NSCLC) continues to claim millions of lives worldwide. Although its poor prognosis is largely attributed to the lack of adequate and precise detection technologies, cancer cells’ suppression of the immune system adds on to the difficulty of identifying abnormal NSCLC tumors in their early stages. Therefore, cancer immunotherapy, which activates the immune system and helps it fight tumors, has recently become the most sought‐after technique, especially in the advanced stages of NSCLC, where surgery or chemotherapy may or may not bring about the desired survival benefits in patients.

**Methods:**

This review focuses on the various immunotherapeutic interventions and their efficacy in advanced NSCLC clinical trials. Monoclonal antibodies like anti‐PD‐1/PD‐L1 agents and anti‐CTLA‐4 antibodies, cancer vaccines, oncolytic viruses and adoptive T cell therapy have been discussed in brief. Furthermore, the effects of gender, age, and race on the efficacy of immune checkpoint inhibitors and suggest plausible future approaches in the realm of immuno‐oncology.

**Results:**

Immunotherapy is used alone or in combination either with other immunological agents or with chemotherapy. However, the efficacy of these strategies depends extensively on various demographic variables, as some patients respond perfectly well to immunotherapy, while others do not benefit at all or experience disease progression. By targeting a “hallmark” of cancer (immune evasion), immunotherapy has transformed NSCLC management, though several barriers prevent its complete effectiveness.

**Conclusions:**

All these immunological strategies should be interpreted in the current setting of synergistic treatment, in which these agents can be combined with chemotherapy, radiotherapy, and, or surgery following patient and tumor characteristics to proportionate the best‐individualized treatment and achieve superior results. To better pursue this goal, further investigations on cost‐effectiveness and sex‐gender, race, and age differences in immunotherapy are needed.

## 1 INTRODUCTION

Lung cancer is one of the most common and deadliest cancers in the world. Every year, around 2 million new cases are detected with a 20 percent mortality rate, which exceeds the mortality rates of colon, prostate and breast cancers.^1^, ^2^ Non‐small cell lung cancer (NSCLC) comprises about 85% of all lung cancer cases.^2^, ^3^, ^4^, ^5^, ^6^ The cause of lung cancer has been attributed to various factors: exposure to asbestos, diesel exhaust, ionizing radiation,^7^ hereditary factors,^7^, ^8^ passive smoking,^8^ alcohol consumption,^8^ exposure to aromatic hydrocarbon emissions in air,^8^ and exposure to carcinogens during work, for example, exposure to radiation in mines or in nuclear plants.^8^ Tobacco smoking, however, is the most predominant risk factor.^4^, ^7^, ^9^ Around 85% to 90% of lung cancers are caused by cigarette smoking.^1^ Based on the microscopic structure of tissues, lung cancer is classified into two main types: Small cell lung cancer (SCLC) and NSCLC. According to the WHO, NSCLC has been classified into three main types: adenocarcinoma, squamous cell carcinoma, and large cell carcinoma.^1^ According to Saab et al., NSCLC can be divided into four subtypes: lung adenocarcinoma (LUAD), lung squamous cell carcinoma (LUSC), large cell carcinoma, and bronchial carcinoid tumor.^10^

Lung adenocarcinoma is the most common type of NSCLC, comprising 40 percent of NSCLC cases — and often affects women who are non‐smokers.^10^ Around 25%–30% of all lung cancers are squamous cell carcinomas,^1^, ^11^ while about 5%–10% of lung cancer cases are large cell carcinomas.^1^ The different stages of NSCLC are defined by the following terminologies.^12^

Occult (hidden) stage: Techniques like imaging and bronchoscopy cannot detect cancer at this stage, but the cancer cells can be found in the patient's sputum and bronchial washings. However, the cancer could have spread to other organs of the body.

Stage 0: Abnormal, potentially cancerous cells (adenocarcinoma in situ or squamous cell carcinoma in situ) start proliferating in the lining of the airways and may spread to healthy tissues in the body.

Stage I: Cancer has already formed, but it does not spread to the lymph nodes. Depending on the size of the tumor and the extent of its damage, Stage I is divided into Stage IA and Stage IB.

Stage II: Stage II is divided into Stage IIA and Stage IIB. In Stage IIA, the tumor is larger than 4 cm but less than 5 cm. In Stage IIB, there are two possibilities^1^: The cancer has spread to the lymph nodes and the tumor is at most 5 cm.^2^ The tumor is larger than 5 cm but at most 7 cm in length, but the cancer has not spread to the lymph nodes.

Stage III: This stage is divided into Stage IIIA, IIIB, and IIIC.

Stage IV: This stage has two classifications, namely, IVA and IVB.

Stages IIIA to IV are the advanced or metastatic stages of NSCLC. They involve numerous complications with regard to tumor size, the extent of spread and cancer metastasis.

The lack of appropriate tools and techniques to detect lung cancer in its early stages and the resulting poor prognosis of the disease^10^ can be attributed to why more than 75% of NSCLC cases are detected in the advanced stages (Stages IIIA to IV).^13^ Over 50% of patients with lung cancer die within a year of diagnosis, while the 5‐year survival rate is approximately 18%.^14^

**FIGURE 1:**
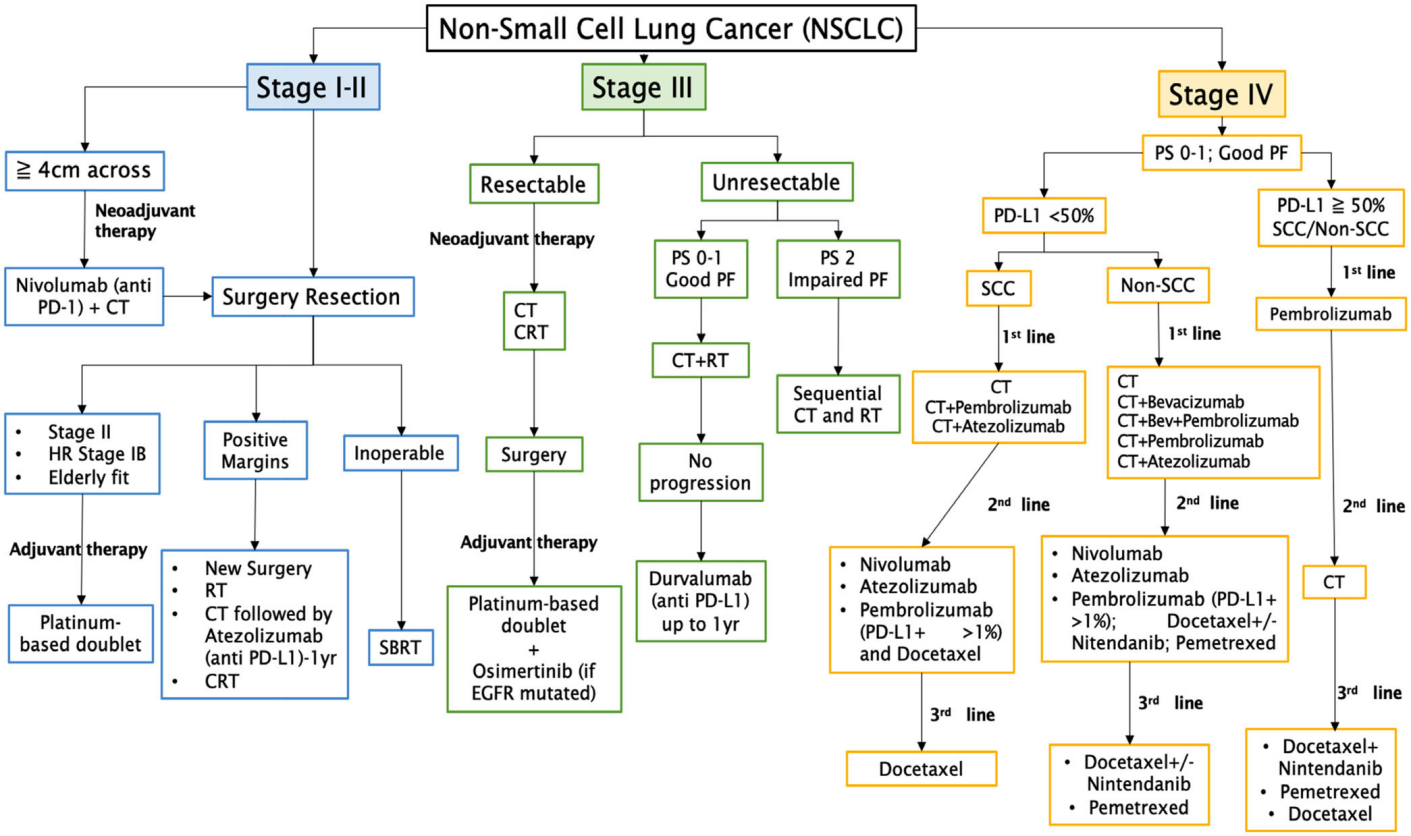
Algorithm that summarizes current NSCLC treatment. Simplified treatment algorithm adapted from the Spanish Society of Medical Oncology (SEOM),^18^ American Society of Clinical Oncology (ASCO),^19^ and American Cancer Society^20^ guidelines. The algorithm does not show data about stage IV patients with PS 2 and PS 3–4, who are treated with carboplatin‐based CT or single agent CT and best supportive care, respectively. High‐risk (HR) Stage IB patients include those with lymphovascular invasion, poor differentiation, or high‐standardized uptake value on positron emission tomography. CT, chemotherapy; CRT, chemoradiotherapy; NSCLC, non‐small cell lung cancer, PF, pulmonary function; PS, performance status; RT, radiation therapy; SBRT, stereotactic body radiation therapy; SCC, squamous cell carcinoma; Osimertinib is a tyrosine kinase inhibitor for EGFR‐mutated NSCLC.

In stages 0, 1, and 2, lung tumors can generally be excised surgically. For Stage IIIA NSCLC, surgery can be used to remove the tumor, depending on the patient's ability to withstand the surgery. Chemotherapy is usually given after surgery to eliminate any remaining cancer cells, so as to increase the patient's survival.^14^ It is difficult to cure Stage IIIB tumors by surgery alone. Depending upon the patient's health and the site of the tumor, initial chemotherapy, a combination of chemotherapy plus radiation therapy, or radiation therapy alone have the potential to be curative.^15^ However, most Stage III lung cancer patients are not allowed to undergo surgery, due to a variety of comorbidities.^16^ In Stage IV, the cancer has metastasized, making surgery a less viable option. Palliative chemotherapy with platinum‐based combination chemotherapy has been the standard treatment for Stage IV NSCLC.^17^ Finally, immunotherapy drugs play a major role in the treatment of advanced NSCLC in Stages III and IV. Figure 1 illustrates a simplified algorithm for NSCLC treatment.^18^, ^19^, ^20^

The immune system can potentially recognize abnormal cells and eliminate them. Tumors can adopt several mechanisms to thwart the immune system. One way is by inducing an immunosuppressive microenvironment locally. Cancer cells can also upregulate the inhibitory immune checkpoints, which, under normal conditions would have prevented the immune system from destroying its healthy cells.^10^ Furthermore, cancer cells could disrupt T cell lymphocyte proliferation and effector functions like cytotoxicity and cytokine release.^21^ Furthermore, malignant cells can cause lymphocytes (T helper, T cytotoxic, T regulatory cells, B lymphocytes and NK cells)^9^ to overexpress multiple inhibitory receptors^21^ like the PD‐1 (Programmed cell death‐1) ligands: B7‐H1 (PD‐L1) (CD274) and PD‐L2 (CD273, B7‐DC).^9^ The PD‐1/PD‐L1 (Programmed cell death ligand‐1) interaction causes T cell dysfunction^22^ and a subsequent suppression of the immune system.^9^ Even effector cytotoxic T lymphocytes (CTLs), which, by their specificity for antigens, can normally induce apoptosis in cancer cells,^23^, ^24^ can be suppressed by tumors. T‐cell subsets (e.g., CD4+ CD25+ Treg cells^25^) also may be recruited by cancer cells to produce immunosuppressive cytokines and express CTLA‐4 (cytotoxic T‐lymphocyte‐associated protein 4).^23^, ^26^ Tumors may also secrete Interleukin 10 (IL‐10) and transforming growth factor β (TGF‐β) to thwart the CTLs from attacking them^26^

Cancer immunotherapy harnesses the body's immune system to launch an immune response against cancer cells. Immune agents like cytokines, vaccines, cell therapies, humoral and transfection agents are used to tweak the immune system.^27^ This review focuses on the various immunotherapeutic interventions and their efficacy in advanced NSCLC clinical trials. Monoclonal antibodies like anti‐PD‐1/PD‐L1 agents and anti‐CTLA‐4 antibodies, cancer vaccines, oncolytic viruses and adoptive T cell therapy have been discussed in brief. It also talks about the effects of various demographic variables (age, gender, and race) that could affect immune checkpoint inhibitor treatments. It also points out certain future approaches that could be adopted to enhance the efficacy of immunotherapy in advanced/metastatic NSCLC

## 2 IMMUNOTHERAPEUTIC AGENTS

### 2.1 Monoclonal antibodies

Immune checkpoint inhibitors (ICIs) like anti‐PD‐1 (nivolumab and pembrolizumab), anti‐PD‐L1 (atezolizumab, avelumab and durvalumab) and anti‐CTLA‐4 (ipilimumab and tremelimumab) antibodies have come a long way in improving a patient's progression‐free (PFS) and/or overall survival (OS) during advanced stages of NSCLC. Targeting PD‐1, PD‐L1, and CTLA‐4 helps the T cell population target and eliminate tumor cells.^28^ Radiologically, one can determine whether an anticancer treatment is effective or not by looking at the size of the tumor. It must be noted that since immunotherapy targets the immune system and not the tumor itself, the time taken to shrink the tumor can vary from individual to individual. Hence, while in some patients the size of the tumor grows up to a particular period and then starts decreasing, some patients (<2%) demonstrate a temporary pseudo‐progression because inflammatory cells infiltrate tumors and physically suggest that the tumor has increased in size. However, this does not mean that the treatment has failed. After an examination in which disease progression is observed, a person can undergo a short‐term follow‐up examination 4 weeks or later to find out whether a pseudo‐progression has occurred, provided his/her condition does not deteriorate.^28^

#### 2.1.1 PD‐1 and PD‐L1 inhibitors

When PD‐1 (present on T cells and other immune cells) interacts with its ligands PD‐L1 and PD‐L2 (present on tumor cells), the number of receptors on the surface of T cells decreases, making the T cells insensitive to cancer cells. Many lung cancers overexpress PD‐L1 in order to downregulate the T cell response.^29^ The expression of PD‐L1 in NSCLC tissues serves as an important biomarker which could help one select an appropriate intervention that will reduce the overexpression of PD‐L1 in tumors.^30^ Immunohistochemistry (IHC) is a sensitive and specific assay that uses specific antibodies (here PD‐1 and PD‐L1 inhibitors) to bind to their respective targets.^31^ PD‐1 inhibitors block the interaction of PD‐1 with PD‐L1 and PD‐L2, but not the interaction of PD‐L1 with CD80 (B7.1).^29^ Anti‐PD‐L1 antibodies block the interaction of PD‐L1 with PD‐1 and CD80 (B7.1), but allow PD‐L2 to interact with PD‐1 and CD80 to interact with CTLA‐4.^29^

##### Pembrolizumab

The Dako PD‐L1 IHC 22C3 pharmDx assay is an immunohistochemistry (IHC) assay approved by the US Food and Drug Administration (FDA). This assay utilizes pembrolizumab (Keytruda) for the diagnosis of advanced NSCLC.^30^ It is robust, sensitive, precise and safe to use on cancer patients.^32^ Developed by Merck Sharp & Dohme,^6^ pembrolizumab is a fully humanized IgG4 kappa isotype monoclonal antibody.^29^, ^33^ For patients with metastatic NSCLC having a PD‐L1 expression of ≥50%, this antibody has been approved by the FDA as the standard first‐line treatment.^3^, ^34^ The antibody can also be used as a second‐line treatment for patients with a TPS (Tumor Proportion Score) ≥1%.^6^ Whether pembrolizumab can be used alone to treat patients with a PD‐L1 expression of <50% still needs to be ascertained.^3^ The common side effects of this antibody include pneumonitis, colitis, thyroiditis,^3^ fatigue, pruritus, and decreased appetite.^33^ To treat such immune‐related adverse events (iRAEs), one can either discontinue pembrolizumab therapy or take corticosteroids.^3^

##### Nivolumab

The US FDA has approved the Dako PD‐L1 IHC 28–8 pharmDx, an IHC assay using nivolumab (Opdivo) as a PD‐L1 inhibitor in metastatic NSCLC.^30^ Nivolumab (BMS‐936558) is a fully human immunoglobulin G4 antibody, developed by Bristol‐Myers Squibb.^29^, ^35^ The first‐line trials in NSCLC patients for nivolumab were not superior to platinum‐based chemotherapy as compared to pembrolizumab.^3^ It is, however, commercially available as a second‐line treatment for advanced squamous and non‐squamous NSCLC,^29^ in patients with a TPS ≥1%.^6^ Nivolumab is used when the first‐line treatment using platinum‐based chemotherapy or treatment using pemetrexed or erlotinib^36^ cannot control disease progression, irrespective of PD‐L1 expression.^29^

##### Atezolizumab

The IHC assay approved by the FDA that uses atezolizumab (Tecentriq) is VENTANA PD‐L1 (SP142).^30^ The assay is reproducible and can precisely detect PD‐L1 expression in TC (tumor cells) and IC (immune cells) in NSCLC.^37^ Atezolizumab (MPDL‐3280A) is a humanized IgG1 monoclonal, antagonistic, anti‐ PD‐L1 antibody^3^, ^29^ that was developed by Roche.^6^ It is used as a second‐line treatment for metastatic NSCLC, in patients with a TC ≥50% or IC ≥10%.^6^ Randomized IMpower150 and IMpower130 trials were carried out on chemotherapy‐naïve patients suffering from non‐squamous advanced NSCLC, and demonstrated that a combination of atezolizumab, bevacizumab, carboplatin and paclitaxel (ABCP) or a combination of atezolizumab, carboplatin and nab‐paclitaxel (ACnP) could be used as first‐line treatment for such patients. However, these trials also had their own iRAEs. In IMpower150, the patients treated with ABCP experienced rash, hepatitis, hypothyroidism, hyperthyroidism, pneumonitis, and colitis. In the IMpower130 trial, the common side effects were rash, hypothyroidism, and hepatitis.^38^

Atezolizumab has also been proven to be beneficial in squamous NSCLC when used in first‐line treatment along with platinum‐based chemotherapy. As compared to docetaxel, atezolizumab was proven to improve the OS when given to patients whose NSCLC (irrespective of PD‐L1 expression) had previously never been treated.^3^, ^11^

##### Durvalumab

The VENTANA PD‐L1 (SP263) IHC assay, approved by the US FDA, uses durvalumab.^30^

Developed by Astrazeneca,^6^ durvalumab (MEDI4736) is a high‐affinity, humanized IgG1κ antagonistic, PD‐L1 inhibiting antibody.^29^ Like nivolumab, durvalumab also failed to showcase benefits over platinum‐based chemotherapy when used in first‐line trials as compared to pembrolizumab.^3^ When first checked for its efficacy in an ongoing phase I/II clinical trial, iRAEs like fatigue, reduced appetite, and diarrhea as well as a fatality of pneumonia were reported. In the ATLANTIC trials, like the previous phase I/II trials, durvalumab showed a durable response in terms of increased PFS in patients who were pretreated. iRAEs in this trial included fatigue, hypothyroidism, asthenia, nausea, and diarrhea.^39^ PACIFIC, the randomized and placebo‐controlled phase 3 trials of this antibody showed that when durvalumab was used in Stage III NSCLC patients with unresectable tumors (but with no progression of cancer after chemotherapy), the PFS of patients was notably extended.^40^ The threshold TPS of such patients must be ≥1%.^6^

#### 2.1.2 CTLA‐4 inhibitors

CTLA‐4 is another inhibitory receptor that disrupts T cell function. It uses CD28, a co‐stimulatory receptor to reduce T cell signaling and suppresses the immune system.^41^ CTLA‐4 inhibitors activate T cells and help them launch an immune response against tumors. They have been shown to be effective in various tumor models in mice and have also been observed to exert a synergistic anti‐tumor response when combined with vaccines, chemotherapy, and radiation.^42^

##### Ipilimumab

Bristol‐Myers Squibb developed ipilimumab (Yervoy) an IgG1 monoclonal antibody.^29^ It was the first anti‐CTLA‐4 antibody that received approval for cancer victims.^41^ However, it failed to bring about the desired effects in NSCLC patients.^29^ Furthermore, a first‐line NSCLC CA184‐041 phase II study by Tomasini et al. used carboplatin/paclitaxel chemotherapy along with ipilimumab. The therapy was tried on advanced NSCLC patients whose tumors were previously untreated. The immune‐related progression‐free survival (irPFS) of the patients improved, with median irPFS of 5.68 months as compared to chemotherapy alone, which prolonged the survival chances upto 4.63 months. Hypophysitis, enterocolitis, and hyperthyroidism were among the main side effects observed.^43^ In order to evaluate how effective the combination of ipilimumab and chemotherapy as a first‐line treatment in advanced squamous NSCLC was, Govindan et al. carried out a randomized, double‐blind, phase III study on patients with stage IV or recurrent chemotherapy‐naive squamous NSCLC. Govindan et al. found that, compared to chemotherapy alone, the OS of these patients who took this therapy did not improve.^44^

##### Tremelimumab

Tremelimumab (CP‐675206)^45^ is a humanized monoclonal antibody developed by AstraZeneca.^29^ Despite having a mechanism of action similar to that of ipilimumab, it has, as of now, not been efficacious when used alone in patients with NSCLC.^46^ It can however be combined with other therapies to treat NSCLC patients.

#### 2.1.3 Comparison between different immune checkpoint inhibitors

Passiglia et al. performed a meta‐analysis and indirectly compared the antibodies nivolumab, pembrolizumab and atezolizumab in pretreated NSCLC patients with respect to various factors and found that nivolumab and pembrolizumab are associated with a significant increase of ORR as compared to atezolizumab and nivolumab is associated with a significant lower incidence of G3‐5 AEs as compared to the other drugs. These evidences could support the oncologists to select the best drug for each patient.^47^

#### 2.1.4 Combination immunotherapy

##### Nivolumab and ipilimumab

Hellmann et al. carried out CheckMate 227 ‐ an open‐label, phase III trial‐ on patients suffering from advanced (Stage IV) or recurring NSCLC (squamous or non‐squamous). Those having a PD‐L1 expression of ≥1% were divided into three equal groups and each group was randomly given the following respective treatments: nivolumab combined with ipilimumab, only nivolumab and only chemotherapy. Patients who had <1% PD‐L1 expression were also divided into three groups, with each group getting the respective treatments: combination of nivolumab and ipilimumab, nivolumab with chemotherapy and only chemotherapy. None of the patients in any of the groups had received chemotherapy before. Results indicated that irrespective of PD‐L1 expression, the first‐line therapy of nivolumab and ipilimumab prolonged the median overall survival (OS) than chemotherapy alone, in patients with PD‐L1 expression ≥1% as well as in those with an expression <1%.^47^ However, a study by Courtney et al. showed that these drugs were far more costly than chemotherapy and, considering the high rates of incidence of advanced NSCLC, the use of combination immunotherapeutic agents could have devastating effects on the economy.^48^

##### Durvalumab and tremelimumab

Rizvi et al. conducted the MYSTIC open‐label phase III randomized clinical trial (RCT) to check whether durvalumab (alone or combined with tremelimumab) was more effective than chemotherapy in the first‐line treatment of metastatic NSCLC. Advanced NSCLC patients with no EGFR (Epidermal Growth Factor Receptor) or ALK (Anaplastic Lymphoma Kinase) genetic alterations were monitored for 3 years. They were divided into three equal groups, with each group getting the respective treatments^1^: Durvalumab monotherapy,^2^ Combination of durvalumab and tremelimumab and^3^ Platinum‐based doublet chemotherapy. The trial did not show any improvement in the OS with durvalumab as compared to chemotherapy. Patients with ≥25% TCs and expressing PD‐L1 did not experience an improvement in either the OS or PFS with the combination therapy versus chemotherapy.^49^

Since disease progression after the first and second‐line treatment is common among some patients with metastatic NSCLC, Planchard et al. conducted ARCTIC ‐ a phase III, randomized, open‐label clinical trial – on heavily pretreated advanced NSCLC patients, to investigate whether the combination of durvalumab and tremelimumab was more effective as compared to the Standard of Care (SoC) chemotherapy for the third‐line treatment of NSCLC. The ARCTIC trial involved two separate studies on two different groups: (A) Durvalumab monotherapy for patients expressing PD‐L1 and having TCs ≥25% and (B) Combination therapy of durvalumab and tremelimumab for patients with TCs < 25% expressing PD‐L1. The researchers concluded that the OS and PFS of patients improved considerably with durvalumab monotherapy as compared to SoC. OS and PFS for those given the combination therapy showed numerical improvements compared to those given SoC chemotherapy.^50^

The POSEIDON (NCT03164616) study, a randomized, open‐label Phase III trial was carried out by Johnson et al. to compare the efficacy of durvalumab with/without tremelimumab combined with different chemotherapy treatments (as per the choice of the investigator) in the first line treatment for metastatic NSCLC (squamous or non‐squamous). Treatment‐naïve patients with EGFR/ALK wild type Stage IV NSCLC were split into three equal groups and given the respective treatments: (A) Durvalumab + chemotherapy followed by durvalumab until the cancer progressed, (B) Durvalumab and tremelimumab along with chemotherapy, followed by durvalumab until cancer progression, with an additional dose of tremelimumab after chemotherapy and (C) Only chemotherapy. The various options for chemotherapy were as follows: platinum + pemetrexed for patients with non‐squamous NSCLC, platinum + gemcitabine for those with squamous NSCLC or carboplatin + nab‐paclitaxel for patients with either histology. Results showed that PFS and OS showed statistically significant improvements with the combination of durvalumab, tremelimumab and chemotherapy as compared to chemotherapy. Combination of durvalumab and chemotherapy showed a marked improvement in PFS as compared to chemotherapy. However, although OS showed a positive trend, it was not statistically significant. Safety profiles for all the three types of treatments mentioned above were similar. The study concluded that durvalumab + tremelimumab + chemotherapy had a potential as a first‐line treatment for Stage IV NSCLC.^51^

##### Pembrolizumab and ipilimumab

Gubens et al. conducted the KEYNOTE‐021 study, a phase 1/2 trial that evaluated the effectiveness of the combination of pembrolizumab and ipilimumab as a second‐line therapy in patients previously treated for advanced NSCLC. Results indicated that the combination drug showed considerable antitumor activity. However, the therapy also led to various side effects in patients such as fatigue, hypothyroidism, diarrhea, pruritus, colitis, pancytopenia, large‐intestine perforation, and diabetic ketoacidosis.^52^

#### 2.1.5 Adverse effects of ICIs

ICIs can give rise to various iRAEs, the occurrences of which depend on various factors such as the time for which the patient has been receiving the drug, the dose administered as well as the antibodies themselves. A patient's genetics as well as the kind of cancer he/she is suffering from can also play a role in the severity of the side effects. Also, the organs that are ultimately affected by the cancer will determine when these iRAEs will be first noticed.^53^ The excessive activation of the immune system can cause it to attack various organs like the kidneys, nervous system, liver, eyes, and even the endocrine system.^54^ The use of ICIs can also give rise to various autoimmune diseases or activate existing autoimmune diseases. Durieux et al. identified many autoimmune mechanisms which led to renal, rheumatic, hematologic, cutaneous, vascular, and neurologic complications during lung cancer, irrespective of the histology.^55^ Some of the adverse conditions can be so life‐threatening that discontinuing the treatment, taking corticosteroids for a long period of time to counter these effects or at times undergoing an anti‐tumor necrosis factor therapy becomes necessary.^54^, ^56^

Su et al. evaluated the risks of pneumonitis and pneumonia caused by ICIs, through a systematic review and meta‐analysis. They concluded that the risk of all‐grade pneumonitis was enhanced in the case of PD‐1/PD‐L1 inhibitors. Although ipilimumab increased the risk of pneumonitis in patients treated with PD‐1/PD‐L1 inhibitors, it did not do so when used as a monotherapy. The rise in the risk of pneumonia after being treated with a PD‐1/PDL‐1inhibitor or CTLA4 inhibitor or a combination of both was not noticeably high.^57^ A meta‐analysis by Hu et al. also found that patients treated with PD‐1/PDL‐1inhibitors had a much higher risk of contracting pneumonia than those treated with chemotherapy. In addition, their examination also unearthed a number of other rare but life‐threatening adverse effects including cardiorespiratory arrest, pancreatitis, severe skin reactions, sepsis, pulmonary embolism, encephalitis, sarcoidosis, endophthalmitis, and myasthenia gravis. Some patients, although few in number, passed away due to some of these rare iRAEs.^58^

#### 2.1.6 Effects of demographic variables on NSCLC immunotherapy using immune checkpoint inhibitors

##### Gender

Through a systematic review and meta‐analysis of phase III RCTs, Grassadonia et al. examined the impact of gender on the survival of advanced NSCLC patients who were treated with ICIs. They concluded that the use of CLTA‐4 inhibitors gave more benefit to men as compared to women in terms of marked improvements in OS and PFS. Their results were in alignment with previous studies which had shown that the use of PD1/PD‐L1 inhibitors may be more effective in males as compared to females, since levels of PD‐L1 expression was demonstrated to be higher in men than in women.^59^ Since the complexity of an immune response can vary in both genders, women may need a therapy other than ICIs alone in order to gain more benefit from immunotherapy than men. With this view in mind, Conforti et al. performed 2 meta‐analyses: (A) RCTs which compared a combination of anti‐PD‐1/anti‐PD‐L1 agents and chemotherapy with chemotherapy and (B) RCTs of first‐line systemic treatment for advanced NSCLC that used PD‐1/PD‐L1 inhibitors either alone or along with chemotherapy. They investigated the efficacy of each of these therapies on both genders. It was found that female patients with advanced NSCLC derived a statistically significant benefit from the combination therapy used in case (A).^60^

Wang et al. studied 15 RCTs involving ICIs on advanced NSCLC patients. Both men and women showed an improvement in OS and PFS. Men experienced this improvement with both PD‐1/PD‐L1 inhibitor monotherapy as well as their combination with chemotherapy. Women, on the other hand, had improved OS but not PFS with PD‐1 inhibitors or monotherapy, and improved PFS but not OS with the combination therapy or PD‐L1 inhibitor therapy. Anti‐CTLA‐4 treatment had no notable effect on either the males or the females. Overall, men seemed to respond more favorably to ICI therapy compared to women.^61^

Certain analyses on the role of gender did not yield any conclusive results. For example, the meta‐analysis by Lai et al. found no role played by gender on the responses to ICI treatment. Compared with conventional agents, the treatment improved the OS and PFS for both men and women in NSCLC as well as in other types of cancers studied.^62^ Similarly, a study by Kapoor et al. also showed that ICIs involved the same levels of efficacy and toxicity in both genders.^63^ Recent studies and their data have shown that β‐estradiol (E2), the female sex hormone, is involved in tumor formation in NSCLC, its prognosis and a patient's response to cancer treatment. The role of estrogen in the interaction between tumors and cells in the vicinity of the tumor microenvironment could pave way for precision‐medicine methods in lung cancer immunotherapy.^64^ Since women are more susceptible to autoimmune disorders, their chances of experiencing more severe adverse effects and their subsequent discontinuation of immunotherapy are larger.^65^

##### Age

Huang et al. conducted an analysis to find an association between age and ICI therapy in cancer patients. They deemed 34 studies as fit for quantitative analysis. Of these, 13 studies involved NSCLC patients. It was found that ICI therapy improved the OS and prolonged the PFS in those <65 and ≥ 65 years of age. The same trend was observed among those aged <75 years. However, it could not be concluded confidently that the therapy was ineffective in patients ≥75 years, because the number of such patients included in the trials was minimal.^66^

Ironically, although patients aged 65 years and above are the ones most commonly afflicted by cancer, the clinical trials that eventually led to the approval of various ICIs like pembrolizumab, nivolumab, and so forth, did not include many patients in that age group and no particular clinical trials have been organized to include only adults over 65 years of age till date.^67^

Lichtenstein et al. retrospectively evaluated NSCLC patients treated with anti‐PD‐1/PD‐L1 agents for 4 years. The age of the patients ranged from ≤60 years to ≥80 years. Although the OS and PFS benefits of immunotherapy differed by age, the study found that rates of toxicity are similar regardless of age.^68^ It was also found that irrespective of age, rates of toxicity were similar for all age groups. A study by Zhang et al. and Li and Gu showed that although the ICI therapy was effective in patients <65 years, it failed to exhibit the desired efficacy in those 75 years of age or older.^68^, ^69^

Although numerous studies continue to compare and evaluate the effectiveness of various ICIs on younger and older adults, while carrying out the actual trials, one must carefully consider the existence of different co‐morbidities and the extent of treatment‐related toxicities in the elderly population before commencing their treatment.^67^

##### Race

Peravali et al. studied patients with Stage IV solid tumors, who were treated with anti‐PD‐1/PD‐L1 antibodies for 5 years. This retrospective study did not include those who were on anti‐CTLA‐4 treatment. The commonly found iRAEs were thyroid stimulating hormone (TSH) elevation, dermatological and rheumatologic. Among elderly patients who had low levels of PD‐L1 expression, low lactate dehydrogenase (LDH) levels and more number of ICI therapy cycles, White patients had 60.4% of iRAEs, while Black patients suffered from 30.8% of the total iRAEs documented. Finally, the data demonstrated a positive correlation between White patients and OS.^70^ According to Nazha et al., the impact of racial differences in various treatments of advanced NSCLC has not been studied in depth because patients from minority groups were not adequately represented in all the crucial clinical trials. The results of the clinical trials of advanced NSCLC patients (median age 69 years) who were treated with single ICIs (nivolumab, pembrolizumab and atezolizumab) were retrospectively analyzed. The patients were divided into various groups and each group received either first‐line, second‐line or third‐line ICI treatment. Improvements in OS and PFS were similar in both Black and White populations and the ORRs for each of them were 15.2% and 23.1%, respectively. The multivariate analysis found no significant association between race and OS/PFS.^71^

An observation by Lee et al. of the clinical data obtained from various ICI treatments of patients with lung cancer stated that the response rates and survival outcomes of Asians were similar to those of the patients from the West, irrespective of the differences in genetics and other profiles. The levels of iRAEs were higher in Japanese patients, but this trend was seldom observed among other Asian patients.^72^ In a meta‐analysis, Peng et al. reviewed seven RCTs of Stage IIIB and IV NSCLC patients with no EGFR mutation who had undergone ICI therapy. Of all patients involved in these trials, 32% were Asians and 68% non‐Asians. The team found no significant difference in the improvements in OS and PFS for both groups of patients. In trials where ICI therapy was being used as a first‐line treatment, a statistically significant higher PFS was found among East Asians than among non‐East Asians during the subgroup analyses. No such trends were seen in case of OS, or in subgroups that received pure ICI therapy or its combination with chemotherapy.^73^

An antibiotic treatment (AT) can interfere with the functioning of the healthy microbial flora in the intestine and thus hamper the efficacy of ICIs. In view of this, Ruiz‐Patiño et al. conducted a retrospective cohort study of advanced NSCLC patients from three different countries in Latin America. These patients were either on anti‐PD‐L1 monotherapy or its combination with other plausible therapies. The team categorized these patients into the following categories^1^: Those never exposed to AT,^2^ Those who received AT within 30 days of receiving dose of the ICI therapy and^3^ Patients receiving the AT along with the ICI treatment. The median OS observed for each of the above categories was 40.6 months, 20.3 months and 24.7 months respectively. The team of scientists did not notice any significant differences in either the PFS or the response rates among any of the categories. The final conclusion was that AT resulted in reduced OS in Hispanic NSCLC patients who were on ICI treatment.^74^

### 2.2 Cancer vaccines

Cancer vaccines help the immune system recognize and remember the antigens expressed by cancer cells, so that it can effectively launch a response against tumors and eliminate them. As of now, the number of preventive vaccines exceeds the number of curative vaccines, and among them are vaccines that help build a patient's immunity against the forms of the human papillomavirus and the Bacillus Calmette‐Guérin (BCG) vaccines.^75^, ^76^ Racotumomab had been approved as a maintenance therapy after a primary treatment for advanced NSCLC in Argentina and Cuba.^75^

A meta‐analysis by Dammeijer et al. showed that the OS and PFS of NSCLC patients increased by using tumor vaccines and cellular immunotherapies. However, cellular immunotherapy was far more effective than the vaccines^77^. Brunsvig et al. developed UV1 (Ultimovacs ASA), an hTERT (human telomerase reverse transcriptase) peptide therapeutic cancer vaccine and attempted to evaluate its safety and patient tolerability through an open‐label, single‐center, dose‐finding phase I clinical trial on patients with Stage III/IV (locally advanced/ metastatic) NSCLC. Prior to the trial, the patients in question had been treated by palliative radiotherapy and/or at least not more than one line of platinum‐based doublet chemotherapy. After conducting the trials with 3 different doses of the vaccine (100, 300, and 700 μg), the researchers concluded that UV1 could initiate an immune response in a large population and that 700 μg was the optimal dose of the vaccine. UV1 could also be combined with ICIs for complete efficacy of the vaccine.^78^ Xing et al. conducted a phase I monocentric, open label dose‐escalation trial to check the efficacy of Hu‐rhEGF‐rP64k/Mont, a therapeutic vaccine for Stage IIIB/IV NSCLC patients. It was observed that the vaccine fostered the desired immunogenic response and was also safe for use in NSCLC patients. The vaccine was approved by the CFDA.^79^

Cuba, Peru, and Venezuela have approved the CIMAvax Epidermal Growth Factor vaccine for Stages IIIB and IV NSCLC when the disease progresses after a first‐line chemotherapy.^75^ The efficacy of this vaccine was monitored in an open‐label, multicentric Phase III clinical trial, in which the patients had already undergone 4–6 cycles of chemotherapy. They were to receive either the vaccine or best supportive care. Those treated with at least four doses of the CIMAvax‐EGF vaccine showed a significant improvement in OS.^80^

A recent development is the Gene‐mediated cytotoxic immunotherapy (GMCI), which uses aglatimagene besadenovec (AdV‐tk), an “armed” non‐replicating adenoviral (Ad) vector (type 5 adenovirus). This is an in situ vaccination method which, when administered, kills cancer cells by inducing apoptosis, autophagy and necroptosis, through pathogen‐associated molecular patterns (PAMPs) that have portions of the viral capsid and viral nucleic acids. Predina et al. carried out a phase I dose‐escalation trial of i.t. neoadjuvant GMCI in NSCLC patients. Their tumors were resected 3 weeks after the vector was injected into them. The GMCI method was found to be both safe and practicable. There was also a noticeable activation of the anti‐tumor immune response.^81^

Dendritic cell (DC) based vaccines are also under investigation. The ability of DCs to take up, process and present antigens makes them the key regulators of the adaptive immune response and hence suitable for making vaccines.^82^ Their advantage lies in the fact that they do not depend on the antigen presenting cells (APCs) present within the body. The effectiveness of these vaccines can be enhanced if they are combined with chemotherapy, radiotherapy and/or checkpoint inhibition. Clinical trials for DC vaccines are still underway and the proper development of this therapy depends on various factors which include the dose of the vaccine, how often must they be injected into the patient and for how long must the sufferer receive the vaccine. The selection of an appropriate combination therapy involving DC vaccines is also of utmost importance to prolong patient survival.^83^

Overall, while many cancer vaccines provide the opportunity to both prevent and treat cancer, have been shown to be well‐tolerated and have the potential to be utilized as a combinatorial therapeutic, they have struggled to show improved OS and PFS, among other survival variables, when used as a singular treatment. In order to continue to improve the efficacy of cancer vaccines, deeper investigation into the biomarkers that underly certain forms of NSCLC, and can subsequently be targeted by cancer vaccines, is needed.^75^

### 2.3 Oncolytic viruses

These attenuated viruses can help launch a de novo immune response or boost the already existing native immune response, thus destroying tumor cells.^84^ The first oncolytic virus approved by the China Food and Drug administration for cancer therapy was a recombinant human adenovirus type 5 named Oncorine (H101). Zhang et al. highlighted a particular incident in which a 57‐year‐old Chinese woman with recurrent NSCLC had developed immune resistance to nivolumab. A combination of Oncorine, nivolumab and anlotinib helped to reverse this immune resistance.^85^

Various oncolytic viruses like the vesicular stomatitis virus (VSV), measles virus (MV), vaccinia virus (VV) and adenovirus (Ad) are being investigated for thoracic cancers. For example, a phase II trial used reovirus in combination with standard chemotherapy in patients with an activated epidermal growth factor pathway in NSCLC. Although the objective response rate was higher than with chemotherapy alone, the results of the trial did not match those obtained from experiments on animal models, which had indicated a synergistic effect of the reovirus‐chemotherapy combination.^86^

Scientists are currently exploring the Newcastle Disease Virus transfection to adjust the genetic transcription of interferon beta (IFNβ) in those lung cancer cells with decreased amounts of IFN.^84^ The measles virus, vaccinia virus and vesicular stomatitis virus have been engineered to produce IFNβ. Despite various tests of these viruses in laboratory NSCLC models, none of these has entered any clinical trials at present.^86^ Recently, recombinant vaccinia viruses (VV) have also been shown to be useful in NSCLC, although not as a monotherapy. Combining TG4010 (MVA‐MUC1‐IL2) with first‐line chemotherapy has proven to be of clinical importance to advanced NSCLC patients. An anti‐tumor immunogenic response can also be launched using STING activation in Batf3‐dependent dendritic cells (DC) using replication‐attenuated VV vectors.^87^

### 2.4 Adoptive T cell therapy

This approach involves growing a patient's T cells in large amounts and genetically engineering them, so that they can express receptors that can help them identify and eliminate cancerous cells. Also called chimeric antigen receptor (CAR‐T) T‐cell therapy, this approach is being currently tested in clinical trials for its efficacy.^76^ China and the United States lead the entire world in terms of CAR‐T trials.^88^, ^89^ For lung cancer, epidermal growth factor receptor (EGFR), human epidermal growth factor receptor 2 (HER2), mesothelin (MSLN), prostate stem cell antigen (PSCA), mucin 1 (MUC1), carcinoembryonic antigen (CEA), tyrosine kinase‐like orphan receptor 1 (ROR1), PD‐L1 and CD80/CD86 are currently being evaluated as potential targets for the CAR‐T therapy.^89^ Feng et al. carried out a phase I clinical study (NCT01869166), in which escalating doses of CAR‐T cell infusions were given to patients with >50% expression of EGFR. The patients were also known to have relapsed/refractory NSCLC. Some common adverse reactions exhibited by the patients included mild skin toxicity, nausea, vomiting, dyspnea, and hypotension. The therapy was proven to be safe and feasible for those with EGFR‐positive advanced elapsed/refractory NSCLC.^90^ A study by Chen et al. showed that transcription factors like NR4A prevented T cells from infiltrating and eliminating solid tumors, and that cancer immunotherapy using CAR‐T cells would be effective if NR4A was inhibited.^91^

Somatic tumor mutational burdens give rise to cancer neoantigens, which are expressed by tumors but not by healthy cells. Robertson et al. suggest that heterogeneous solid tumors like NSCLC could possibly have clonal neoantigens as the most pertinent targets for cancer immunotherapy. A major problem in developing a clonal neoantigen‐reactive T cell (cNeT) product is that one must easily detect clonal neoantigens. Secondly, the T cells of the product must be capable of proliferation and persistence with time, and this is possible only through an appropriate scale‐up process during the product's manufacture. Moreover, the functional effector phenotype of T cells should remain conserved in vivo. One such cNeT product is ATL001, whose safety and activity in NSCLC patients is currently being evaluated in a Phase I/IIA trial called ATX‐NS‐001 (CHIRON).^92^

## 3 DISCUSSION

Immunotherapy represents an exciting new frontier in the world of NSCLC treatment. PD‐1 and PD‐L1 inhibitors, such as Pembrolizumab, Nivolumab, Atezolizumab, and Durvalumab have been FDA authorized as treatments for advanced and/or metastatic NSCLC.^29^, ^30^, ^32^, ^37^ CTLA‐4 inhibitors, which similarly work to activate T cells to jumpstart an anti‐cancer cell immune response, have shown promise when employed in concert with cancer vaccines, chemotherapy, and radiation.^42^ Furthermore, the usage of both PD‐1/PD‐L1 and CTLA‐4 inhibitors together has also been a growing point of investigation, although concerns exist about the cost‐effectiveness and marginal benefits of this approach.^49^ Cancer vaccines and oncolytic viruses have also been evaluated and implemented as treatment methods for NSCLC. Cancer vaccines, when used with immunotherapy, have been shown to improve OS of NSCLC patients, while oncolytic viruses boost the native immune response and have shown relative efficacy when used with chemotherapy.^78^, ^85^, ^88^ Finally, adoptive T cell therapy has offered a novel approach to immunotherapy, where a patient's T cells are cultured and engineered to target and kill cancerous cells.^77^ Table 1 highlights the benefits and challenges of these therapies.

**TABLE 1:** Assessment of benefits and challenges of immunological agents for NSCLC treatment

Treatment	Benefits	Challenges
Pembrolizumab (PD‐1 inhibitor)	Robust, precise, safe to use^32^ Approved as the first line of treatment for patients with PD‐L1 expression >50%^3^	Ability to be utilized to treat patients with PD‐L1 expression of less than 50 percent is unknown^3^ Variety of immune‐related adverse events, such as pneumonitis, colitis, and thyroiditis^3^
Nivolumab (PD‐1 inhibitor)	A viable third option when platinum‐based chemotherapy or pemetrexed/erlotinib are unsuccessful, regardless of PD‐L1 expression^29^, ^36^ Can be utilized as a second‐line treatment for both advanced squamous and nonsquamous NSCLC when tumor proportion scores exceed one percent^6^, ^29^	Faltered in trials as a first line treatment when compared to pembrolizumab and platinum‐based chemotherapy^3^
Atezolizumab (PD‐L1 inhibitor)	Well established as a second‐line treatment option where either more than 50 percent of tumor cells or 10 percent of tumor‐inflitrating immune cells have PD‐L1 expression^6^ Can be utilized as a second‐line treatment for both advanced squamous and nonsquamous NSCLC when tumor proportion scores exceed one percent^6^ Has potential as a first‐line combination therapeutic either with another immunotherapeutic or with platinum‐based chemotherapy^3^, ^11^, ^38^	Variety of iRAEs have been documented following use, such as rash, hepatitis, hypothyroidism, hyperthyroidism, pneumonitis, and colitis.^38^
Durvalumab (PD‐L1 inhibitor)	Has shown increased, durable progression‐free survival (PFS) times during clinical trials^39^ Phase 3 trials have shown that durvalumab can extend PFS for Stage III NSCLC patients with unresectable tumors^40^	Struggled to show benefits over platinum‐based chemotherapy and pembrolizumab as a first‐line treatment^3^ Variety of iRAEs^39^
Ipilimumab (CTLA‐4 inhibitor)	A phase 2 study demonstrated that, in concert with carboplatin/paclitaxel as a first‐line combinatorial therapeutic for advanced NSCLC, immune‐related progression free survival is better than that for just chemotherapy^43^	Overall survival is not improved for Stage IV or chemotherapy‐naïve squamous NSCLC patients following combined chemotherapy and ipilimumab treatment^44^ Hypophysitis, enterocolitis, and hyperthyroidism are three of the main side effects^43^
Tremelimumab (CTLA‐4 inhibitor)	Has shown promise as a combinatorial therapeutic^47^	Not efficacious when used as a singular therapeutic for NSCLC^46^
Nivolumab and ipilimumab (Combination therapy)	Has been shown to prolong overall survival when compared to chemotherapy alone regardless of PD‐L1 expression^47^	Incredibly more costly than chemotherapy^48^
Durvalumab and tremelimumab (Combination therapy)	Has shown potential as a third‐line therapeutic when compared to the standard of care^50^ The combination of durvalumab, tremelimumab, and chemotherapy for Stage IV NSCLC patients has the potential to be a first‐line treatment^51^	A phase III trial indicated that there are no improvements to the overall survival of metastatic NSCLC patients receiving durvalumab and tremelimumab when compared to those chemotherapy as a first‐line treatment^49^
Pembrolizumab and ipilimumab (Combination therapy)	As a second‐line treatment, this combination has shown antitumor activity in phase 1 and phase 2 trials^52^	A plethora of side effects are associated with this combination, including fatigue, diabetic ketoacidosis, colitis, pruritis, and large‐intestine perforation^52^
Cancer vaccines	Can safely be combined with chemotherapy, radiotherapy, and/or checkpoint inhibition in order to improve effectiveness^83^ Dendritic cell vaccines in specific do not depend on antigen‐presenting cells in the body to function^83^ Can be tooled to either prevent cancer formation, or treat an existing cancer^75^	Cellular immunotherapies have often outperformed tumor vaccines in effectiveness^77^ Many vaccines have struggled to demonstrate response benefit in terms of improved OS and PFS^75^
Oncolytic viruses	Can address immune resistance to certain immunotherapies^85^ Can be combined with chemotherapy, leading to improved response rates and the potential for synergism^86^	Doubts surrounding their potential to be utilized as a monotherapy^87^
Adoptive T cell therapy	Has been shown to be safe despite mild adverse reactions^90^ Following the inhibition of certain transcription factors, CAR‐T cell therapy has the potential to be an effective method of solid tumor elimination^91^	Manufacturers must have the ability to scale‐up the generation of T cells^92^ Concerns surround the ability of the T cells to maintain their functional effector phenotype in vivo^92^

Vaccine	Approval status	Overview
Bacillus Calmette‐Guérin (BCG)	Approved for non‐muscle‐invasive bladder cancer for over 40 years^105^	Standard of care for high‐risk, non‐muscle‐invasive bladder cancer Has been shown to decrease risk of recurrence compared to alternative treatment methods (transurethral resection, intravesical chemotherapy)^105^
Sipuleucel‐T (Provenge)	FDA‐approved in 2010 for asymptomatic or minimally symptomatic metastatic castration‐resistant prostate cancer (CRPC)^106^	Autologous cellular vaccine that utilizes dendritic cells programmed to identify prostatic acid phosphatase Has been shown to prolong median survival by four months Current investigations have focused on evaluating the use of Sipuleucel‐T in concert with other CRPC therapies^106^
T‐VEC (Imlygic)	FDA‐approved for unresectable metastatic stage IIIB/C–IVM1a melanoma^107^	First cancer vaccine containing an oncolytic herpes virus (herpes simplex virus 1) approved by the FDA for unresectable melanoma Has shown strong efficacy as both a monotherapeutic and a combinatorial therapy in concert with immune‐checkpoint‐inhibitors^107^
UV1	Designated as an orphan drug by the FDA for stage IIB‐IV melanoma^108^	Targets human telomerase reverse transcriptase (hTERT), which is present in approximately 85% of all solid tumors^78^ Is being evaluated for efficacy in Phase I and II trials across numerous cancers, including melanoma, non‐small cell lung cancer, ovarian cancer, and head and neck cancer^108^
CIMAVax	Approved in Cuba, Venezuela, and Peru for Stage IIIB and IV NSCLC^75^	Approved for use following the completion of a first‐line chemotherapeutic^75^ Utilizes a human recombinant epidermal growth factor (EGF) in order to stimulate an immune response against autologous EGF^109^ Recent trials have demonstrated efficacy in lengthening the median overall survival of NSCLC patients with stable disease^110^

Novel immunotherapies	
Immunotherapy	Trial status and results, if published
FLT3 Ligand (CDX‐301) with stereotactic radiotherapy^111^	Currently in Phase II trials to evaluate progression‐free survival following success in murine models, with an estimated study completion date of October 5, 2022
Sacituzumab Govitecan‐hziy^112^	Currently in a Phase III, multi‐center trial (recruiting phase) to evaluate efficacy versus Docetaxel to address advanced or metastatic NSCLC following platinum‐based chemotherapy and anti‐PD‐1/PD‐L1 immunotherapy Estimated date of study completion is January 2025
High‐activity natural killer (HANK) cell^113^	Phase I and II trial to evaluate the use of HANK cell transfusions to address small metastases of NSCLC, specifically through the evaluation of relief degree of tumors, progress free survival, and overall survival Study recruitment completed in September 2019
T‐Cell Receptor Therapy (TCR‐T)^114^	Utilization of tumor‐responsive T cells obtained from tumors that are subsequently cloned and reinfused to target NSCLC tumors Will evaluate dose‐limiting toxicity, complete and partial remission, and TCR‐T cell persistence Long‐term study with an estimated study completion date in 2033
IMU‐201 (PD1‐Vaxx)^115^	Goal is to generate a polyclonal induced B‐cell antibody response through a vaccine that delivers B‐cell and T‐cell epitopes Phase I study that includes dose‐escalation, response rate, overall/progression‐free survival, and immunogenicity evaluation Currently in the recruitment phase
T‐cell Receptor Immunotherapy with Chemotherapy^116^	Phase II study Combination of T‐cell receptor therapy, chemotherapy, and interleukins (Aldesleukin) in order to shrink NSCLC tumors Will evaluate patient clinical response rate, the characteristics of tumor‐infiltrating lymphocytes and potential adverse events Estimated completion date is in October 2025
ADXS‐503 (with or without pembrolizumab)^117^	ADXS‐503 is a Listeria‐based immunotherapy that includes *Listeria monocytogenes* engineered to elicit various T cell responses against NSCLC tumor antigens Phase I and II trial to evaluate the tolerability and anti‐tumor activity of ADXS‐503, and the overall and progression‐free survival of patients Currently recruiting patients
TG4010^118^, ^119^	TG4010 is a therapeutic vaccine that includes a modified poxvirus engineered with MUC1 (a tumor‐associated antigen) and interleukin 2 Phase II and Phase III trials to evaluate TG4010 with chemotherapy in advanced NSCLC patients were completed in July of 2014 TG4010 with chemotherapy was found to improve progression‐free survival compared to chemotherapy alone The most common grade 3–4 adverse events as outlined by the National Cancer Institute Common Toxicity Criteria were found to be generally similar
TF2^120^	Phase I and II trial to evaluate the use of anti‐carcinoembryonic antigen radioimmunotherapy (anti‐CEA RAIT), wherein a bi‐labeled antibody (bsMAb) targets NSCLC tumors and delivers radiation Two specific goals of determining the optimal TF2 protein dose for the radiation delivery (IMP‐288‐Lutetium) and the maximum tolerated dose of the imaging (IMP‐288‐Indium) and treatment radiation Were able to successfully demonstrate low immunogenicity and low toxicity of the anti‐CEA RAIT, and the potential for the anti‐CEA RAIT to be utilized as an anti‐tumor therapeutic^121^
BMC128 with nivolumab^122^	Phase I, first‐in‐human, proof‐of‐concept trial of BMC128, a selection of parts of the gut microbiome, in combination with nivolumab, an immunotherapeutic Current trial has not started to recruit participants as of April 2022 Key outcome measures include the number and severity of drug‐related treatment emergent adverse events, objective response rate, clinical benefit rate, and duration of response
Inupadenant (EOS100850) with chemotherapy^123^	Will be studied as a second line treatment for metastatic and stage III nonsquamous NSCLC Part 1 of the study focuses on the optimal dose of the drug with carboplatin and pemetrexed Part 2 compares the efficacy of inupadenant to a placebo given with carboplatin and pemetrexed Will have 192 participants with an estimated completion date of May 2025

Beyond the particularities of various NSCLC immunotherapies, however, lie the effects of various demographic variables, which emphasize the need for precision medicine when creating immunotherapy plans for NSCLC patients. For example, men have been demonstrated to respond more positively to ICI treatment, while women have been shown to benefit from joint immunotherapy and chemotherapy.^60^, ^61^ Furthermore, the underrepresentation of certain minority groups in clinical trials of immunotherapeutics has hampered the ability for researchers to truly evaluate racial and cultural differences in receipt of and response to immunotherapy.^72^

In spite of data showing consolidated findings regarding sex differences from NSCLC tumorigenesis^64^ to immunotherapy response,^60^, ^61^, ^64^ females are still underrepresented in clinical trials assessing the impact of immunotherapy.^93^ Sex‐gender differences in tumor features, hormone fluctuations, immunological pathways, metabolization rates, and others are variables that can impact treatment outcomes, and then clinical results should not be extrapolated from one sex to another. While these differences are best known for ICIs,^59^, ^60^, ^61^ we still lack information on how sex variability can impact cancer vaccines, oncolytic viruses, and adoptive T cell therapies. In addition, the gay, bisexual, lesbian, transgender, and intersex populations also need more attention from future trials.^94^, ^95^ As the transgender population increases,^96^ the implications that gender‐affirming hormone therapy and social determinants of health may have on immunotherapy effectiveness should be better evaluated.^94^, ^95^, ^96^, ^97^ Moreover, since sexual chromosomes can have several roles in immune response and regulation,^93^ intersex patients should also receive more attention based on their chromosome status. Consequently, upcoming studies and clinical trials should consider addressing these questions in their designs to guarantee a better‐personalized approach for the future.

Identification of appropriate biomarkers for NSCLC will greatly help optimize immunotherapy for the same. Biomarkers can be tissue based (PD‐L1 expression, Tumor Mutational Burden [TMB], Tumor‐infiltrating lymphocytes [TILs], etc) or serum based (neutrophil‐to‐lymphocyte ratio [NLR], platelet‐to‐lymphocyte ratio [PLR], Blood tumor mutational burden [bTMB], etc).^98^ Early stages of NSCLC could be detected by liquid biopsy (LB), a minimally invasive test^99^ that involves looking for cancerous cells or DNA pieces from tumors in blood.^100^ Mildner et al. reviewed various publications and for convenience considered tests done on blood, urine and other body fluids under LB. Based on their analysis, they came up with some more potential biomarkers for advanced NSCLC immunotherapy: Cell free DNA (cfDNA) found in human plasma, circulating tumor DNA (ctDNA) from tumors, peripheral blood mononuclear cells (PBMCs), circulating tumor cells (CTCs), soluble mediators found in plasma and serum, exosomes and microRNA.^99^ Nevertheless, the absence of an ideal biomarker and cut‐off still narrows the LB use for translational research. As these techniques become more accurate and standardized, they will likely be incorporated into clinical routine, favoring immunotherapy efficiency and a better precision medicine approach.^101^

Although immunotherapy (alone or along with chemotherapy) has revolutionized the field of oncology, a significant proportion of the population does not benefit from it and experiences disease progression due to various resistance mechanisms adopted by their bodies against these therapies. Horvath et al. have listed immunogenicity, immuno‐adaption, regulatory T cells (Treg) and myeloid‐derived suppressor cells (MDSCs), the four major subgroups of chemokines namely CC, CXC, CX3C, C as well as vascular endothelial growth factor (VEGF) as some of the possible immune resistance mechanisms. Strategies like using Stimulator of Interferon Genes (STING) agonists like STING‐binding molecules and cyclic dinucleotide (CDN) derivatives along with ICIs therapy can effectively launch an anti‐tumor response by making tumors sensitive to ICIs.^102^

Moreover, with the growing popularity of personalized medicine, CAR‐T cell therapy is an exciting candidate for the generation of treatments that are targeted toward a particular patient's cancerous cells.^77^ Within the next decade, the expansion of CAR‐T cell therapy to engineer more novel chimeric antigen receptors to identify a broader range of surface proteins on cancer cells and to decrease the toxicity of the therapy will enable it to reach a wider population of NSCLC patients. Although the results are promising, the potential for increases in health spending can cause financial concern. For example, administering CAR‐T to all indicated patients with non‐Hodgkins lymphoma would increase the US health spending on this disease by 68%.^103^ Nonetheless, CAR‐T could provide more incremental quality‐adjusted life‐years than the average pharmaceutical and nonpharmaceutical interventions, while retaining similar cost‐effectiveness.^104^ Besides that, due to its recent approval, long‐term outcomes compared with other treatments are not yet available, which gives a lot of uncertainty to these estimations.^103^ Therefore, a precise CAR‐T therapy cost‐effectiveness estimation for NSCLC patients involves a lot of challenges. The same issues may apply to other innovative strategies, such as cancer vaccines and oncolytic viruses.

Ultimately, a careful consideration of the interplay of various variables like genetics, lifestyle and emotional well‐being of a person, a thorough estimation of the expenses incurred for experiments on animals as well as on humans and an appropriate, unbiased selection of patient population for clinical trials, and the recognition of the growth of precision medicine will go a long way in providing a better quality of life for patients with advanced NSCLC, and the further advancement of cancer immunotherapy in the next decade.

## 4 CONCLUSION

ICIs are the immunological agents that have changed NSCLC's natural history. They can also act as adjuvants when combined with chemotherapy and other immunotherapies (see Table 1), increasing current treatment effectiveness. Although all this progress, there are still patients insensitive or that acquire resistance to this strategy. As several mechanisms can be related to this ineffectiveness, the development and consolidation of LB will make it more likely to screen patients for prognostic factors and biomarkers, allowing for better clinical decisions and ICI outcomes. Oncological viruses could be a potential therapy to target this resistance, and cancer vaccines could prevent its acquisition or help with its treatment. CAR‐T cell therapy is also a new approach that could improve these outcomes by adding limited toxicity.

By targeting a “hallmark” of cancer (immune evasion), immunotherapy has transformed NSCLC management, though several barriers prevent its complete effectiveness. Therefore, all these immunological strategies should be interpreted in the current setting of synergistic treatment, in which these agents can be combined with chemotherapy, radiotherapy, and, or surgery following patient and tumor characteristics to proportionate the best‐individualized treatment and achieve superior results. To better pursue this goal, further investigations on cost‐effectiveness and sex‐gender, race, and age differences in immunotherapy are needed.

## AUTHOR CONTRIBUTIONS

**Gayatri Iyer:** Conceptualization (equal); data curation (lead); investigation (lead); methodology (supporting); writing – original draft (lead); writing – review and editing (equal). **Akhil Rekulapelli:** Conceptualization (supporting); methodology (supporting); project administration (supporting); writing – original draft (supporting); writing – review and editing (lead). **Lucas E. Flausino:** Data curation (supporting); methodology (supporting); writing – original draft (supporting); writing – review and editing (lead).

## CONFLICT OF INTEREST

No funding has been obtained for this review. Dr. Balkrishnan is currently a consultant for Ostuka Pharmaceuticals Inc. None of the other authors have any pertinent conflict of interests.

## ETHICS STATEMENT

This study is a review of published research and is exempt from Human Subjects Board review.

## Data Availability

Data sharing is not applicable to this article as no new data were created or analyzed in this study.
